# Effect of COVID-19 Pandemic on Well Child Care and Vaccination

**DOI:** 10.3389/fped.2022.873482

**Published:** 2022-04-19

**Authors:** Grace Onimoe, Dhanalakshmi Angappan, Marie Christianne Ravie Chandar, Sarah Rikleen

**Affiliations:** ^1^The MetroHealth System, Cleveland, OH, United States; ^2^Case Western Reserve University, Cleveland, OH, United States

**Keywords:** COVID-19, pandemic, well child care visits, vaccination, children

## Abstract

**Objectives:**

The COVID-19 virus is highly contagious primarily via aerosol transmission and has a high mortality rate. On March 13, 2020, the United States declared a national emergency in response to the COVID-19 pandemic. This study aims to enumerate the effect of the pandemic on vaccination rates during the COVID-19 lockdown and the aftermath in pediatric patients aged 6weeks-6 years.

**Study Design:**

A retrospective review of medical records was performed of missed well childcare visits at MetroHealth from March 1, 2020 to June 30, 2020. The sample size of 400 children aged 6 weeks to 6 years were randomly selected. Demographic data, number of calls made to attempt, scheduled WCC, no show rates for clinic appointments, number of missed WCC, location of MH facility, insurance type, vaccination status prior to the pandemic were collected. Statistical analysis was performed with SPSS software (IBM Corp. Released 2020. IBM SPSS Statistics for Windows, Version 27.0. Armonk, NY: IBM Corp).

**Results:**

From this descriptive study, we found that 43.5% of patients were not up to date on their childhood vaccination. The mean age was 24.38 months (SD 20.15). There were slightly more males (52.8%) in the study than females (47.3%) and most children were of African American descent. More than 50% of patients missed a scheduled well child appointment and 27% had a missed at least two consecutive appointments.

**Conclusion:**

The COVID-19 pandemic has no doubt made a significant mark on health care; the effects would be both immediate and delayed, with vulnerable population being the most impacted. There is an urgent need to prevent a large-scale health disaster of catastrophic potential that could occur if an effective vaccination strategy is not implemented rapidly.

## Introduction/Background

The first U.S. cases of non-travel related COVID-19 were confirmed on February 26 and 28,2020. During this time, there was little known scientific information on its virulence and the aftereffects of the illness. The COVID-19 virus was proving to be highly contagious primarily via aerosol transmission and had a high mortality rate. On March 13, 2020, the United States declared a national emergency in response to the Coronavirus (COVID-19) pandemic.

The pandemic changed everyday life in many ways and had a profound effect on health care delivery in the United States. This effect on health care was global, as according to a WHO report published in August 2020, 90% of 105 countries reported at least some disruptions to essential health services, with routine immunization services, among the most frequently disrupted. (1, 2). The pediatric population though impacted, did not experience a high mortality rate. Families heeded warnings from public health officials and kept children inside and home from schools and daycares to limit exposure to the virus. However, strict adherence to home quarantine created a new problem that placed infants and children at a great disadvantage of missed evaluations by medical personnel (3, 4). In a bid to protect children, parents simply stopped showing up for routine well-child care and medical illnesses. This trend continued even when physicians' offices reopened for limited in-person visits. This created a situation where vaccines were deferred. The Boston Medical Center which serves about 14,000 vulnerable patients, 90% of whom are on public insurance, recorded an 80% decrease in clinic volume in March 2020. (3). This scenario was widespread across numerous pediatric practices across the country. Vaccines for Children program (VFC) provides vaccinations to children who are both underinsured and insured. Funding for the VFC program is approved by the Office of Management and Budget (OMB) and allocated through the Centers for Medicare & Medicaid Services (CMS) to the Centers for Disease Control and Prevention (CDC). Vaccines available through the VFC Program are those recommended by the Advisory Committee on Immunization Practices (ACIP)(5). VSD is a collaborative project between CDC's Immunization Safety Office and eight U.S. health care organizations serving publicly and privately insured patients.VSD indicated a notable decrease in orders for VFC- funded, ACIP-recommended vaccines, specifically non-influenza childhood vaccines and measles-containing vaccines during January 6, 2020- April 19, 2020, compared to January 7, 2019–April 21, 2019. (6). The decline began following the declaration of a national emergency by President Trump on March 13, 2020, similar declines in orders for other vaccines were also observed. (6). Worldwide, in a poll of 260 healthcare practitioners in May 2020, respondents in 53(85%) of 61 countries reported lower vaccine levels than those recorded in January and February 2020 (1, 7).

It is important to avoid a disruption in immunizations to prevent communities from vaccine-preventable diseases and outbreaks during the COVID-19 pandemic. Per Vogt et al. study, the results of a current national survey indicated that most VFC-enrolled practices were open and offering routine immunization services to all pediatric patients in May 2020 (8). There was a sharp decline in VFC vaccine orders beginning in March and continuing through April (6), orders during the second half of May 2020 and the first 3 weeks of June were relatively comparable to those from the same period in 2019), suggesting that the current immunization infrastructure can meet the expected need to provide vaccines that are overdue to many children. concerns about access to routine immunization services among certain populations of children, particularly those living in urban areas and the Northeast (8).

This descriptive study aims to enumerate the effect of the pandemic on vaccination rates during the COVID-19 lockdown and the aftermath in pediatric patients aged 6weeks-6 years receiving medical care at Metro Health System's outpatient pediatric primary care clinics from March 1 to October 30th, 2020. A list of missed scheduled well-child visits during a specified period was identified and a retrospective review of medical records was performed. Demographic, categorical, and continuous data were collected and analyzed as depicted below.

## Methods

### Overview

A retrospective review of medical records was performed of missed well childcare visits (WCC) at Metro Health (MH) from March 1, 2020, to June 30, 2020. Among 9,594 children scheduled for WCC, approximately 3,680 missed WCC were identified in children aged 0-18 years. The sample size of 400 children aged 6 weeks to 6 years was randomly selected. Inclusion criteria were pediatric patients receiving medical care at MH hospital before COVID-19 and newborns who initiated care at MH facilities. Patients were excluded if they were >18 years of age, utilizing non-MH facilities for primary care, and did not establish care in Pediatric MH Clinics from March 2020 to June 2020. The primary endpoint was demonstrating the effect that COVID-19 had on childhood vaccination rates via missed clinic visits. Secondary endpoints included identifying locations with the highest number of missed visits and the vaccines/age group most impacted. Results will be utilized to map out a program intervention to increase pediatric childhood vaccination rates.

### Data

Data were then extracted from clinic visits that occurred between March 1 and October 30, 2020. Delayed vaccination was defined as vaccines that were not administered due to a missed scheduled WCC. WCC was defined as an in-person clinical encounter for a routine physical, recommended annually for children aged above 3 years and more frequently for infants and children under 3 years of age.

Demographic data including age, gender, ethnicity, and zip codes were collected.

Continuous variables (number of calls made to attempt, scheduling WCC, no show rates for clinic appointments) and categorical variables [zip codes, number of missed WCC, location of MH facility, insurance status (YES/NO), insurance type, vaccination status before the pandemic—up to date (UTD) and not up to date (NUTD)] were collected. Statistical analysis was performed with SPSS software (IBM Corp. Released 2020. IBM SPSS Statistics for Windows, Version 27.0. Armonk, NY: IBM Corp). Graphical and numerical summaries represented the data collected. Results are expressed as mean ± standard deviation (SD) for normally distributed variables. Chi-Square tests evaluated MH clinic location and zip code about vaccination status during COVID-19. All analysis was two-tailed, and *p*-values < 0.05 were considered statistically significant.

## Results

**Figure 1:**
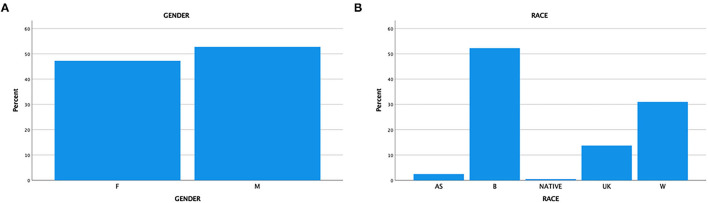
**(A)** Gender. **(B)** Race.

**Figure 2:**
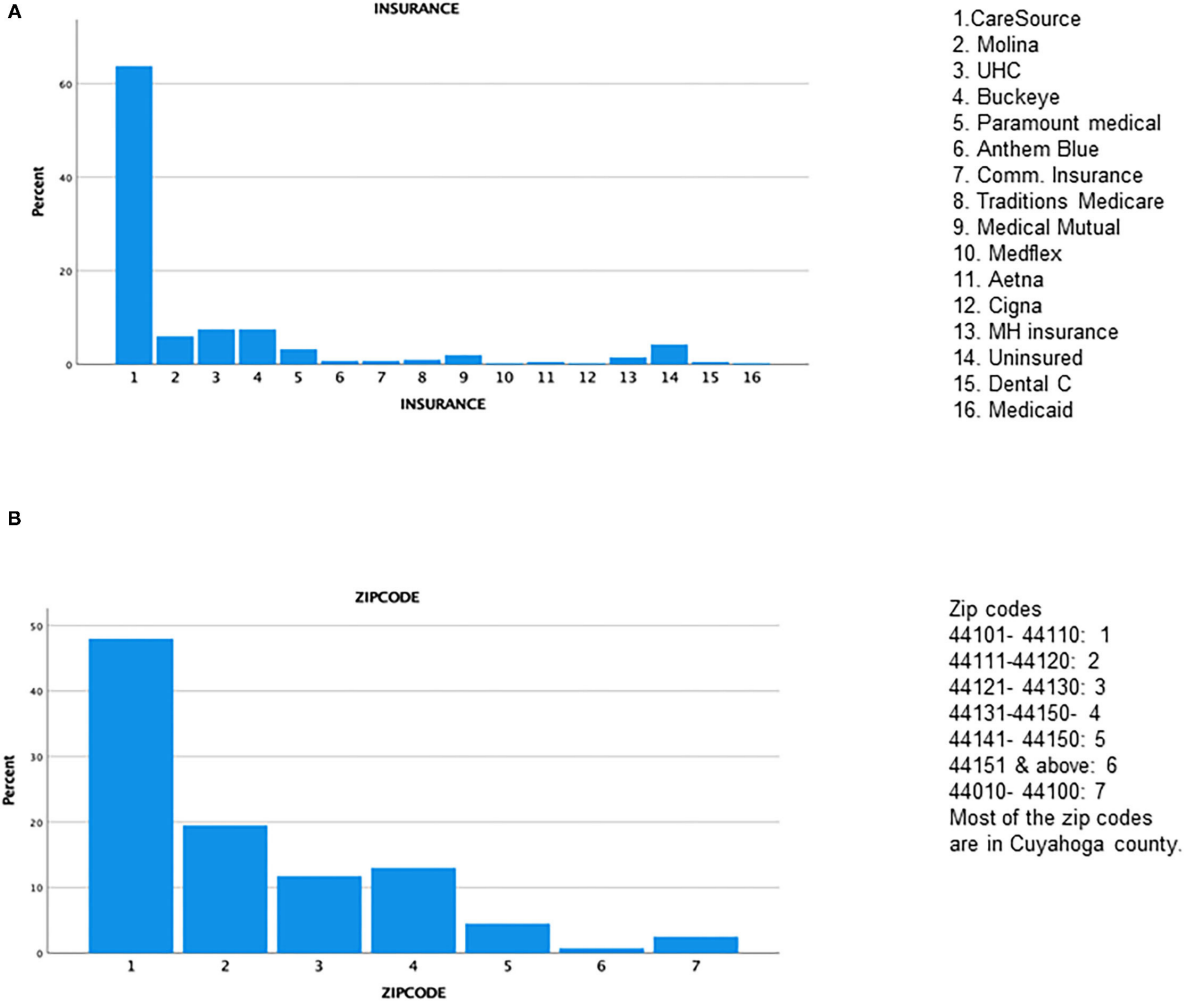
**(A)** Insurance status. **(B)** Zipcode.

Four hundred patients were randomly selected for the study. The mean age was 24.38 months (SD 20.15); a large SD indicates that most individuals' ages are spread out far from the mean. There were slightly more males (52.8%) in the study than females (47.3%) and most children were of African American descent (Figures 1A,B, Table 1). Most patients had public health insurance majorly, 4.3% of patients were uninsured; the near majority (48%) resided in the East Cleveland region (zip code 1; 44101–44110) (Figures 2A,B), which has a poverty rate of 37.51% (9).

**Table 1:** Descriptive statistics *demographics*.

**Variables**	**Total**
	***n* = 100**
**Gender**	
Male	52.8%
Female	47.3%
**Race**	
Asian	2.5%
Black	52.3%
Native	0.5%
Unknown	13.8%
White	31%
**Zip Code**	
44101-44110	48%
44111-44120	19.5%
44121-44130	11.8%
44131-44140	13%
44141-44150	4.5%
44151 and greater	0.8%
44010-44100	2.5%
**Insurance Status**	
CareSource	63.7%
Molina	6%
UHC	7.5%
Buckeye	7.5%
Paramount medical	3.3%
Anthem blue	0.8%
Comm insurance	0.8%
Traditions medicare	1.0%
Medical mutual	2.0%
Medflex	0.3%
Aetna	0.5%
Cigna	0.3%
MH insurance	1.5%
Uninsured	4.3%
Dental C	0.5%
Medicaid	0.3%

**Figure 3:**
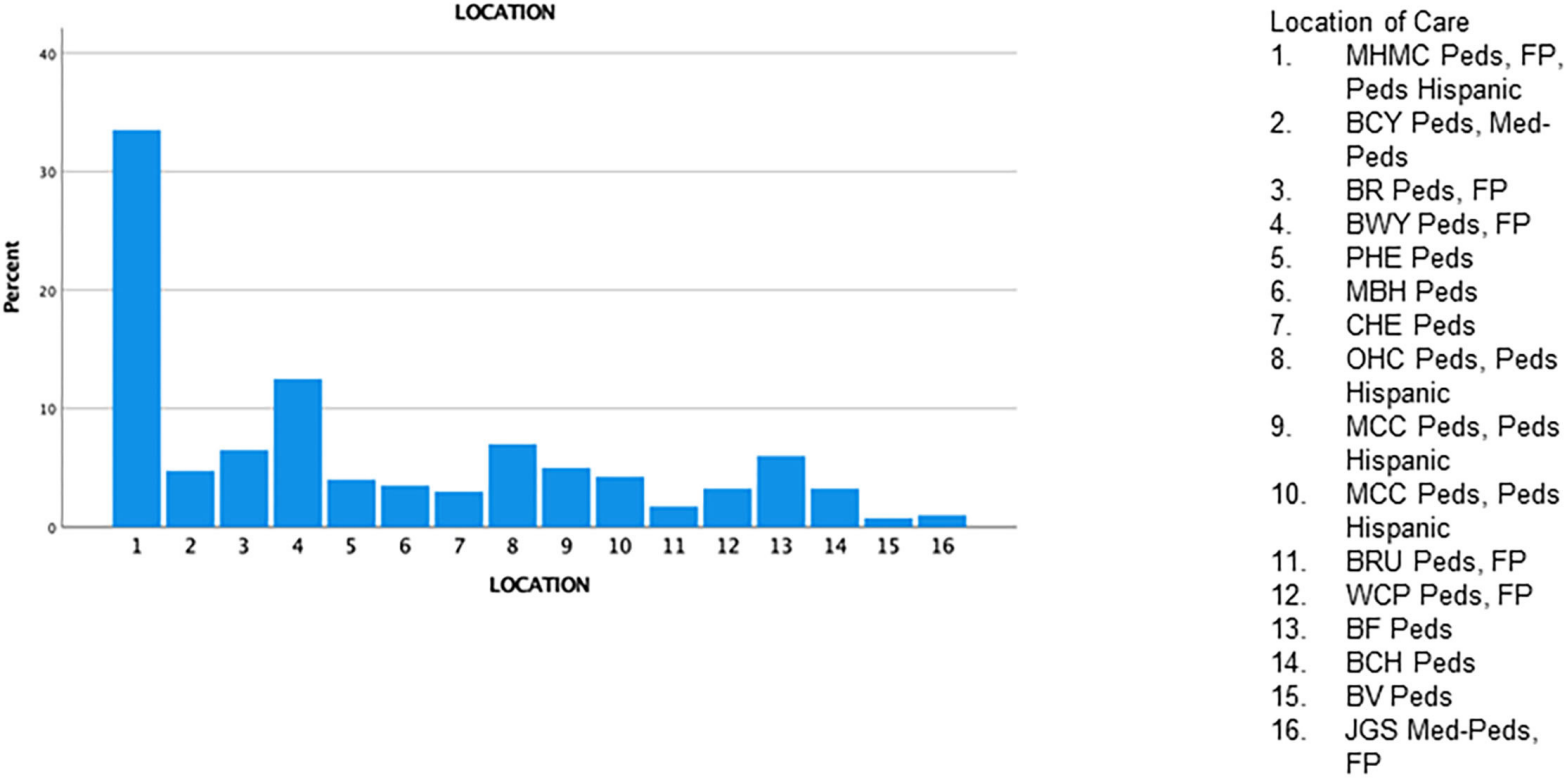
Pediatric clinic locations.

**Figure 4:**
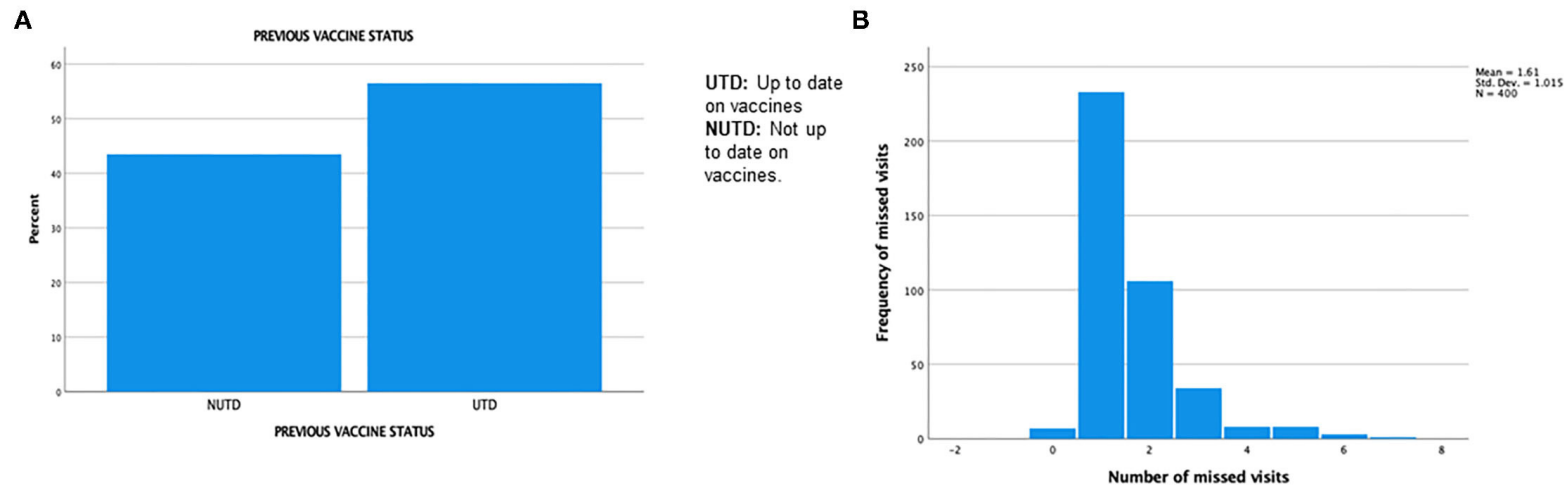
**(A)** Previous vaccine status. **(B)** Number of missed clinic visits.

**Figure 5:**
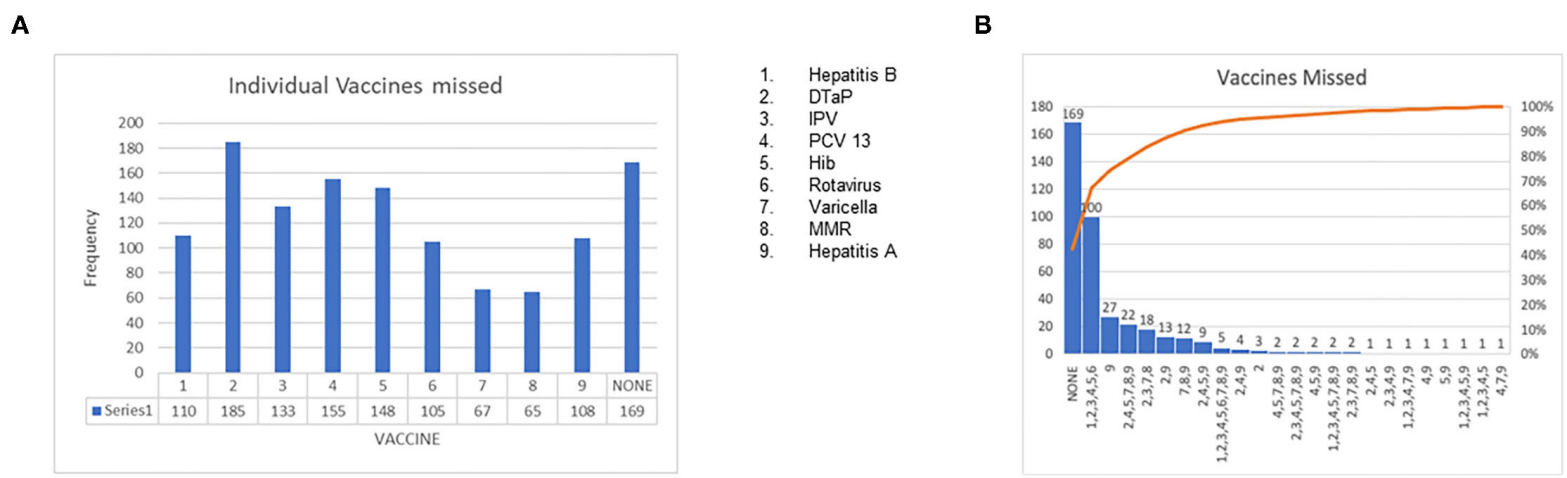
**(A)** Presentation of individual vaccines missed. **(B)** Presentation of all missed vaccines/vaccine combinations.

Of the 16 MH pediatric clinic sites, the main campus (MHMC) was the site where most patients received care (33.5%), 12.5% received care at the Broadway center (Figure 3) and the remaining 15 sites were in the minority. Forty-four percent of patients were NUTD before the scheduled well-child visit in 2020 (Figure 4A). The outpatient service line placed priority in remainders being sent for scheduled visits; there were few documented calls to remind caregivers to schedule appointments, but this does not translate to the calls not being placed. More than 50% of patients missed a scheduled well-child appointment; 27% had missed at least two scheduled visits (Figure 4B). No show rates were a fixed value in electronic medical records (EMR) that did not specifically reflect the study period but rather the overall visits to the facility. The mode for no-show rates was 50%. MHMC clinic site had the greatest counts of missed visits and NUTD patients, however, there was an effort made by caregivers to attend visits as 63.2% of patients were seen after a missed visit. The vaccines that had the highest frequency of being missed were the DTaP while the most common vaccine combination missed was Hepatitis B/DTAP/IPV/PCV 13/Hib/Rotavirus (Figures 5A,B). Interestingly, few vaccination combinations were missed only once or twice, due to study subjects being delayed probably even before the pandemic. Several combinations of the 2, 4, 6-month shots excluded the rotavirus, as the infant was probably beyond the maximum age it could be administered. There was a high frequency of missed optional vaccine hepatitis A although data collection did not distinguish between first and second dose, there was an anecdotal report of parents requesting deferment on the optional vaccine during the pandemic. The results reported a higher-than-expected number of patients that were NUTD in zip codes 1 (MHMC), 3 (Brooklyn), and 4 (Broadway), all areas with median household income lower than the state (Ohio) median income (9). Location 8 (Ohio City) had the highest divergence of the observed from the expected; the higher number of patients receiving care at this location were NUTD (19 patients) than expected (12.2 patients). This contrasted with the most common location for care (MHMC) having nearly equivocal data for observed and expected dichotomous data (NUTD: observed 59; Expected 58.3. UTD: Observed 75; Expected 75.5).

There was no correlation between vaccination status and location (*p* = 0.370), nor was there any correlation between vaccination status and zip code (*p* = 0.122) (Table 2). Previous vaccine status correlated with race; black populations were more likely to be NUTD on vaccinations (*p* = 0.045). There was no correlation between previous vaccine status (UTD/NUTD) and showing up for a clinic visit after a missed appointment (*p* = 0.092).

**Table 2:** Bivariate analysis-categorical outcomes (vaccination status).

**Variable**	**Vaccination**			
		**UTD**	**NUTD**	***P*-value**
		**N = 56.5%**	**N = 43.5%**	
**Location**				
1		56%	44%	0.370
2		57.9%	42.1%	
3		53.8%	46.2%	
4		58%	42%	
5		68.8%	31.3%	
6		78.6%	21.4%	
7		50%	50%	
8		32.1%	67.9%	
9		55%	45%	
10		58.8%	41.2%	
11		85.7%	14.3%	
12		46.2%	53.8%	
13		66.7%	33.3%	
14		46.2%	53.8%	
15		66.7%	33.3%	
16		75%	25%	
**Zip Code**				
44101–44110		53.1%	46.9%	0.122
44111–44120		62.8%	37.2%	
44121–44130		55.3%	44.7%	
44131–44140		48.1%	51.9%	
44141–44150		77.8%	22.2%	
44151 and beyond		100%	0%	
44010–44100		56.5%	43.5%	

## Discussion

The COVID-19 pandemic has un-folded cracks existing in the healthcare industry. Disruptions to healthcare-associated with the COVID-19 pandemic have been well described (10–14). While it is encouraging that 56.5% of patients were UTD on childhood immunizations before the pandemic, it is worrisome that 43.5% were not. Well-child visits are settings in which childhood vaccinations are routinely administered and our study shows a reduction in scheduled visits which translates to interrupted schedules in vaccination of patients. Of note, no-shows were highest with areas with reduced median incomes suggesting the impact of social determinants of health. There was a paucity of documented efforts to contact patients to schedule WCC visits. Though we did not collect data on reasons for missed visits, we found in our clinical practice tele visits that most families communicated that they would not come to the facility due to the ongoing pandemic. Telemedicine though available during this period had the obvious limitation of inability to perform physical examinations or administer vaccines.

Our study mirrors findings abroad, as an international study showed a lower number of children received vaccination in 2020 compared with 2019 ranging from 50 to 85% depending on the individual vaccine analyzed (15). Interestingly, anecdotal reports from this international study noted there were no problems with vaccine supplies in the areas studied due to COVID-19 restrictions, hence the reduced vaccinations were not related to vaccine supplies (15).

Recommendations to increase vaccination status should include mandating calls by identified MH staff to caregivers so they can provide information on missed vaccine status and the importance of complying with clinic visits for vaccinations. During these calls, MH staff should discuss the safety precautions being taken by the facility to limit the risk of virus transmission. Mailed fliers, MyChart messages, and asking the patient/caregiver what mode of reminder works best for their lifestyle can be employed to remind caregivers of scheduled appointments.

Designing vaccination drives to occur at the most impacted locations in a publicly held facility with proper social distancing and sanitization, along with the engagement of community leaders is another strategy of increasing the vaccination rate. Expanding the vaccine drive-through program to occur at the campus that hosts the population with the lowest vaccination status and or sites where a large majority of patients receive care (MHMC), should be enacted. Providing incentives to caregivers that encourage compliance with WCC will be helpful.

Expanding clinic hours through offering evening and weekend clinics at select sites could be beneficial to the cause of catching up on immunizations. In addition, when schools reopen in person, implementing vaccination catch-up booths at schools, after prior consent from parents, will prove helpful for older children. There is a growing need to increase awareness among the public about the need to keep up with vaccinations, through social media and other networking platforms by trusted sources, to prevent the spread of misinformation, dispel myths and regain confidence in the health care system.

Though the majority were on public insurance, there has been monumental job loss resulting in loss of employer-based insurance, and this subset of patients impacted should be identified and offered free vaccination that could potentially be underwritten in cots by the local health department.

Finally, COVID-19 has no doubt made a significant mark on health care, the effects would be both immediate and delayed, with the vulnerable populations being the most impacted. This calls for well-coordinated action and engagement in increasing vaccination rates across various spheres. This study reports important information on vaccination and well-child evaluations during the pandemic in a medically underserved population. Study limitations include possible selection bias and information bias, Unable to directly elicit reasons for missed visits, Study period not an extended duration, and the design method does not include ongoing/future data collection. Additionally, we were also unable to compare no-show rates before the pandemic to current no-show rates as it is only reported as an aggregate of scores by the electronic health records.

In summary, a pandemic is in season, but there is an urgent need to prevent a health disaster of catastrophic potential, which could occur if preventable diseases that are deceased by effective vaccination strategy develop rapidly due to a failure to strategize and increase vaccination rates during a pandemic.

## Data Availability Statement

The original contributions presented in the study are included in the article/supplementary material, further inquiries can be directed to the corresponding author/s.

## Ethics Statement

The studies involving human participants were reviewed and approved by MH IRB. Written informed consent from the participants' legal guardian/next of kin was not required to participate in this study in accordance with the national legislation and the institutional requirements.

## Author Contributions

GO designed research. All listed authors contributed in retrieving data, analyzing data and manuscript writeup. All authors contributed to the article and approved the submitted version.

## Funding

This research study and publication has been supported by John Patrick Carey Foundation of Pediatric Research at The MetroHealth System.

## Conflict of Interest

The authors declare that the research was conducted in the absence of any commercial or financial relationships that could be construed as a potential conflict of interest.

## Publisher's Note

All claims expressed in this article are solely those of the authors and do not necessarily represent those of their affiliated organizations, or those of the publisher, the editors and the reviewers. Any product that may be evaluated in this article, or claim that may be made by its manufacturer, is not guaranteed or endorsed by the publisher.
