# Corrigendum: Effect of COVID-19 pandemic on well child care and vaccination

**DOI:** 10.3389/fped.2022.1014634

**Published:** 2022-08-25

**Authors:** Grace Onimoe, Dhanalakshmi Angappan, Marie Christianne Ravie Chandar, Sarah Rikleen

**Affiliations:** ^1^The MetroHealth System, Cleveland, OH, United States; ^2^Case Western Reserve University, Cleveland, OH, United States

**Keywords:** COVID-19, pandemic, well child care visits, vaccination, children

In the published article, the **Funding** statement was omitted. The correct **Funding** statement appears below.

## Funding

This research study and publication has been supported by John Patrick Carey Foundation of Pediatric Research at The MetroHealth System.

The authors apologize for this error and state that this does not change the scientific conclusions of the article in any way. The original article has been updated.

## Publisher's note

All claims expressed in this article are solely those of the authors and do not necessarily represent those of their affiliated organizations, or those of the publisher, the editors and the reviewers. Any product that may be evaluated in this article, or claim that may be made by its manufacturer, is not guaranteed or endorsed by the publisher.

